# Morphology of the criminal brain: gray matter reductions are linked to antisocial behavior in offenders

**DOI:** 10.1007/s00429-020-02106-6

**Published:** 2020-06-26

**Authors:** Lena Hofhansel, Carmen Weidler, Mikhail Votinov, Benjamin Clemens, Adrian Raine, Ute Habel

**Affiliations:** 1 Department of Psychiatry, Psychotherapy and Psychosomatics, Medical Faculty, RWTH Aachen University, Pauwelsstraße 30, 52074 Aachen, Germany; 2 Institute of Neuroscience and Medicine (INM-10), Research Center Jülich, Jülich, Germany; 3 Departments of Criminology, Psychiatry, and Psychology, University of Pennsylvania, Philadelphia, PA USA

**Keywords:** Aggression, Antisocial behavior, Psychopathy, Voxel-based morphometry, MRI

## Abstract

Aggression and psychopathy are multifaceted conditions determined interpersonal and antisocial factors. Only a few studies analyze the link between these separate factors and specific brain morphology distinctively. A voxel-based morphometry (VBM) analysis was performed on 27 violent offenders and 27 controls aiming to associate sub-features of aggressive and psychopathic behavior with specific gray matter volumes. Trait aggression was assessed using two self-report tests (Aggression Questionnaire, AQ, and Reactive–Proactive–Aggression Questionnaire, RPQ) and psychopathy with the Psychopathy Checklist-Revised (PCL-R). Total and sub-scale scores of these tests were correlated to the brain morphometry of the offenders group in separate analyses. It was found that psychopathic behavior was negatively correlated with prefrontal gray matter volume and that this result was primarily driven by the antisocial behavior sub-scale of the PCL-R. Furthermore, less gray matter in right superior frontal and left inferior parietal regions with increasing antisocial behavior could be observed. One cluster comprising the right middle and superior temporal gyrus was negatively correlated with both, reactive aggression and antisocial behavior. These results outline (1) the importance of distinctively analyzing sub-features that contribute to aggressive and psychopathic behavior, given that the negative correlation of psychopathy global scores with prefrontal volume was driven by one single facet of the PCL-R scale (antisocial behavior). Moreover, these results indicate (2) fronto-temporo-parietal network deficits in antisocial, criminal offenders, with a particular strong effect in the temporal lobe.

**Electronic supplementary material:**

The online version of this article (10.1007/s00429-020-02106-6) contains supplementary material, which is available to authorized users.

## Introduction

Persistent aggressive and psychopathic behavior bare immense burden for the proximate environment as well as monetary costs for the society. Individuals with high psychopathic traits, commit more violent crimes (Glenn and Raine 2009; Hare and McPherson 1984; Hare et al. 1991; Williamson et al. 1987), and have higher rates of criminal recidivism compared to non-psychopaths (Anderson and Kiehl 2014a, b; Harris et al. 1991). The relationship between violent criminal behavior and psychopathy has been studied for decades, revealing strong associations and conceptual overlaps between both constructs (DeLisi 2009). An important similarity is that both aggression and psychopathy can be subdivided into emotional and behavioral components. While psychopathy comprises the prevalence of both, affective and impulsive–antisocial behavior, the two most widely employed dimensions of aggressive behavior include reactive–impulsive and proactive-instrumental aggression. With regard to the different sets of behaviors that contribute to the two main dimensions of psychopathic and aggressive behavior, distinct neural networks and brain regions that amplify these different behaviors are expected to be involved.

Previous studies aiming to determine robust and valid biomarkers of psychopathic or aggressive behavior yielded a complex pattern of results. Several studies reported decreased gray matter volume (GMV) to be associated with increased aggressive behavior, in frontal, temporal, occipital and parietal lobe areas, and more specifically in the dorso- and ventrolateral prefrontal cortex, orbitofrontal cortex, anterior cingulate cortex, lateral and medial parts of the temporal lobe, the temporal poles, insula, postcentral gyrus, fusiform gyrus, superior and inferior parts of the parietal lobes and the cerebellum. On the other hand, positive correlations between GMV and violent behavior were found in the striatum, thalamus and hypothalamus (see Lamsma et al. 2017 for review). Almost identical regions have been negatively linked with psychopathic behavior (e.g. dorso-, lateral-, medial frontal and orbitofrontal cortex, cingulate cortex, insula, temporal regions, parahippocampal and fusiform areas), but again with rather inconclusive results (see Pujol et al. 2018 and Griffiths and Jalava 2017 for review and Koenigs et al. 2011; Pujol et al. 2018; Yang et al. 2009, 2010).

This brief outline of previous results underlines a twofold problem. First, it becomes evident that biomarkers for both psychopathic and aggressive behavior might be undistinguishable or have substantial overlap. This finding can probably be attributed to the difficulty to clearly discriminate between the two constructs. Secondly, there is evidence that both, increases and decreases of regional brain volumes have been linked with psychopathy and aggression. Recent review articles (Griffiths and Jalava 2017; Lamsma et al. 2017; Pujol et al. 2018) attributed this finding to primarily methodological discrepancies between studies. Varying assessment tools (self-report questionnaires vs. clinical interviews) and inconsistent diagnostic cutoffs impede study comparability to the same extent as heterogeneous sample characteristics (forensic groups with or without comorbidities or non-criminal controls). Both Lamsma et al. (2017) and Pujol et al. (2018) showed that, in some of the reviewed studies, psychopathy scores were assessed to describe the sample, but were not included into analyses, whereas trait aggression was not quantitatively acquired by tests, but derived from past behavior (e.g. convictions for violent crimes). Additionally, in prior studies the dimensional characteristics of both constructs were often overlooked in the calculations. Only very few studies implemented regression analysis in their designs, which would allow a direct link between the severity of aberrant behavior and possible biological markers. This problem was further addressed in the review article by Griffiths in 2017, focusing on structural characteristics of global psychopathy. Of the 30 selected studies, 25 were based on group comparisons between psychopaths and non-psychopathic controls and only five studies performed correlation analyses, whereby in two cases this was conducted in a non-forensic group. All of these studies performed hypothesis-driven region of interest analyses and no whole brain approaches.

To overcome the issues encountered by testing complex constructs, behavioral studies have already distinctively analyzed the sub-features that individually contribute to psychopathy and aggression separately and further, focused on their dimensional character. Unfortunately, this approach is still rather sparsely represented in the field of neuroimaging and the few existing studies produce rather inconclusive results.

With the aim to identify brain structure characteristics of psychopathic and aggressive behavior, we set up an experiment to focus in particular on the link of sub-factors contributing to these two constructs with brain morphology. With regard to mentioned methodological issues of previous research, we recruited a violent cohort and assessed both, psychopathy as well as trait aggression with well validated and reliable tests that allowed us to examined respective sub-factors of the two constructs independently. To achieve comparability with previous studies, we recruited a non-criminal control group to allow group comparisons and correlation analyses with the traits of interest in the control cohort with brain morphology.

With reference to the literature, we hypothesized to find GMV reductions in offenders compared to a respective control group in prefrontal, temporal and parietal regions and, therefore, replicate the findings of the previous studies (Bertsch et al. 2013; Contreras-Rodríguez et al. 2015; Gregory et al. 2012). Furthermore, we expected that specific components of aggressive and psychopathic behavior would be associated with distinguishing brain morphology characteristics. Since the first dimension of psychopathy, defined by instrumental, proactive aggression and emotional detachment, could previously be linked to lack of empathy and guilt in addition to low reactivity to stress and punishment, we expected to find structural alterations in brain regions involved in affective components of moral decision making, such as the amygdala, but also in orbitofrontal, anterior cingulate, insula and temporal cortices (Blair 2016; Gregory et al. 2015; Kiehl et al. 2018; Raine 2018). On the other hand, reactive aggression and impulsive–antisocial behavior were highly associated with the inability of offenders to control their behavior and modulate the intensity of their negative responses. Hence, negative correlations of reactive aggression and GMV in ventromedial prefrontal, temporal and the temporo-parietal junction regions involved in behavioral control, response inhibition and cognitive components of empathy were to be expected (Anderson and Kiehl 2014b; Brown et al. 2012).

## Materials and methods

### Participants

Sixty-seven male violent offenders were recruited from three different parole offices in Aachen, Germany. A male control group without any criminal background was recruited by public advertisement. Axis I disorders of all participants were assessed using the German version of the Structured Clinical Interview (SCID) for DSM-IV Disorders (Wittchen et al. 1997). Participants were excluded from the study if they had any acute mood, psychotic or anxiety disorder, if any type of opiates had been taken in the past 12 months, if they exceeded the age range of 18–55 years, and if they held any contraindications for MRI measurements (e.g. metal implants). After application of these criteria, 27 criminal offenders and 28 controls were left eligible for participation in this study. All criminal offenders were convicted for at least one violent crime, such as armed robbery (in 13 cases), assault (eight cases), burglary (two cases), sexual offence (two cases) or manslaughter (two cases). Written informed consent was obtained from each participant and they were financially compensated for participation. This study was conducted in accordance with the Declaration of Helsinki and approved by the Institutional Review Board of the Medical Faculty, RWTH Aachen University.

On the first day of the experiment, all participants underwent comprehensive neuropsychological and psychiatric screenings. MR measurements were performed on a separate day. Data of one participant assigned to the control group had to be discarded due to poor imaging data quality, resulting in a total of 54 participants with 27 in each group.

### Neuropsychological testing

In all participants IQ was estimated by the verbal crystallized intelligence test (WST) (Kose et al. 2015). Trait aggression was assessed using the aggression questionnaire (AQ) by Buss and Perry (von Collani and Werner 2005) and the Reactive–Proactive Aggression Questionnaire (RPQ) (Raine et al. 2006), both in their German versions. The AQ is a 29-item questionnaire in which participants rate on a 5-point scale how characteristic certain statements about themselves are (1—“extremely uncharacteristic of me”, 5—“extremely characteristic of me”). A factor analysis (Buss and Perry 1992) revealed four sub-scales outlining physical and verbal aggression, anger and hostility. The RPQ consists of 23 items that evaluate the occurrence of certain acts of aggression (temper tantrums, vandalism) in the participants past, ranging from 0—“never” to 2—“often” and describes the sub-scales proactive aggression and reactive aggression separately.

Psychopathy scores of criminal offenders were obtained with the German version of the Psychopathy Checklist—Revised (Mokros et al. 2017). As the PCL-R is an instrument developed specifically for forensic cohorts and since the likelihood of psychopathy in the general population is very low, the PCL-R was performed only among the offenders. The PCL-R is a scale based on a semi-structured interview conducted by a trained professional who rates 20 Items on a three-point scale (0 = does not apply; 1 = applies somewhat or 2 = definitely applies). The sum score of the PCL-R ranges from 0 to 40 and reflects the dimension of the participants’ psychopathic traits (Hare et al. 2000). The two-factor model of the PCL-R provides two dimensions of psychopathic behavior: Factor 1 describes interpersonal and affective aspects issues, while factor 2 is defined by antisocial deviant behavior (Hare et al. 1990, 1991). In 2003 the four facet model has been proposed (Hare 2003), according to which factor 1 can be further subdivided into interpersonal problems (facet 1) such as glibness, superficial charm, grandiose self-worth and pathological lying, and specifically affective traits (facet 2) such as lack of remorse, responsibility or guilt, shallow effect and callousness. Factor 2 was divided into a lifestyle facet (facet 3) described by stimulation seeking behavior, impulsivity and lack of realistic goals and an antisocial sub-scale (facet 4) which is composed of items such as poor behavioral control, revocation of conditional release, juvenile delinquency and criminal versatility. According to the manual, an interpretation of the PCL-R-total score is not recommended if more than five items cannot be estimated. If a minimum of three or more items are missing, one should refrain from calculating factor scores, while facets cannot be ascertained if more than two items could not be scored. In consequence, the total score and factor 1 scores could be estimated in 26 offenders; factor 2, facet 2 and 3 in 25 offenders and facet 4 was assessed in 24 offenders.

### MRI data acquisition

Magnetic resonance imaging (MRI) data was collected using a 3-Tesla PRISMA MR scanner (Siemens Medical Systems, Erlangen, Germany) located in the Department of Psychiatry, Psychotherapy and Psychosomatics, Medical Faculty, RWTH Aachen University Hospital. T1-weighted structural images were acquired with a 20-channel head coil by means of a three-dimensional magnetization-prepared rapid acquisition gradient echo image (MPRAGE) sequence (voxel size: 1 × 1 × 1 mm, 256 × 256 matrix, FoV: 256 × 256 mm^2^, 176 slices, TR = 2300 ms, TE = 2.98 ms, flip angle = 9°). The acquisition of T1 images was part of a combined functional MRI-neurostimulation study reported elsewhere (Hofhansel et al. in press).

### MRI data preprocessing

A voxel-based morphometry (VBM) analysis was performed using the CAT12 toolbox (http://www.neuro.uni-jena.de/cat) for Statistical Parametric Mapping Software (SPM12) (https://www.fil.ion.ucl.ac.uk/spm) implemented in Matlab2015b (Mathworks, Inc., Natick, Massachusetts, United States). For preprocessing, we followed the recommendations and defaults stated in the handbook (Gaser and Dahnke 2016) of the toolbox. Standard preprocessing involved four steps. First, tissue segmentation classified gray matter, white matter and cerebrospinal fluid of the individual raw T1-images. In a second step, all images were affine registered to standard tissue probability maps (TPM) by correcting individual head positions and orientations, and translated into Montreal Neurologic Institute (MNI) space. Next, a normalization step corrected for volume changes of the segmented images by applying linear deformation. At last, modulated gray matter images were smoothed with a Gaussian kernel of 8 mm full width at half maximum (FWHM).

### Data analysis

Behavioral data was analyzed using SPSS version 25 (IBM Corp. Released 2017. IBM SPSS Statistics for Windows, Version 25.0. Armonk, NY: IBM Corp.). Scores from neuropsychological tests, questionnaires and estimated brain tissue volumes were compared between both groups using a two-sided student’s *t* test. Tests on the correlations between aggression and psychopathy sub-scales in offenders were performed using bivariate Pearson’s correlation.

Imaging data was analyzed using SPM 12. Group comparison analysis between offenders and controls GMV was performed on a whole brain voxel-by-voxel level. Therefore, the smoothed gray matter segments of all participants were implemented into a full-factorial general linear model (GLM) with factor *group* (two levels) using the theory of Gaussian random fields. To control for individual brain volume variations and the interaction of age and brain volume we entered total intracranial volume (TIV) and age as control variables into the model (Gaser and Dahnke 2016). Since offenders and controls differed in intelligence, we also included individual IQ scores as variable of no interest into the group comparison. Due to the unequal distribution of substance use disorders (SUD) among both groups, and hence to avoid an over-estimation of the group variable, we desisted from including this factor into the statistical model of the group comparison.

To analyze the link between brain morphology and the traits of interest, two different models of analyses were computed. For model 1, separate correlational analyses for each sub-scale of aggression and psychopathy measures were performed in voxel-by-voxel whole brain multiple regression analyses in the offenders group, again using TIV and age as control variables and the respective factor of interest (i.e. reactive aggression) as explanatory variable. Since intelligence levels were coherent within both sample groups, the variable was not implemented into the model. For model 2, all sub-scales of respective tools were implemented into one overall statistical design with again, including TIV and age as covariates. In consequences of limited space, results of model 2 can be found in the supplemental material (S1).

All results were thresholded at a significance level of *p *< 0.001, with a cluster level correction at *p*_(uncorrected)_ < 0.05. To avoid edge effects at the border between gray and white matter, we excluded all voxels with a value of < 0.1 (absolute masking threshold) (Gaser and Dahnke 2016).

Only the regression analyses performed in the offenders group are discussed in the main manuscript. The aggression scores of the controls were located at the bottom of each scale, leading to a lack of variance and emerging floor effects in the analysis which might corrupt the interpretation of these results. However, respective analyses in the control group and the overall entire sample (combined offenders and controls) can be found in the supplemental material (S2 and S3). All results were presented in the MNI space and reported regions were defined by the WFU pickatlas for SPM (Maldjian et al. 2003). The effect sizes of significant clusters revealed by multiple regression analyses were calculated by extracting individuals beta-values of each cluster using the MarsBar toolbox for SPM (http://marsbar.sourceforge.net/), performing partial correlation analyses (using TIV and age as covariates) in SPSS and transforming the correlation coefficient (*R*^2^) into cohens’s *f*^2^ (Selya et al. 2012).

## Results

### Behavioral results

#### Sample characteristics

As depicted in Table 1, the group of violent offenders had less years of education and lower levels of verbal intelligence compared to the control group. Furthermore, higher levels of anger, physical, reactive and proactive aggression were observed in the criminal cohort. No significant group differences were found for age, verbal aggression and hostility.

**Table 1:** Sample characteristics and group comparisons

	Controls (HC)	Offenders (OF)	Statistics	
	*M* (SD)	*M* (SD)	*t* (df)	*p*
*N*	27	27		
Age	34.37 (10.30)	35.59 (9.47)	− 0.454 (52)	0.652
Years of education	14.15 (2.40)	10.44 (0.93)	7.482 (51)	0.000***
Verbal IQ	107.92 (14.55)	95.93 (8.50)	3.662 (50)	0.001**
SUD (*N*)	3.70% (1)	74.07% (20)		
Aggression questionnaire (AQ)				
Physical aggression	17.78 (5.06)	25.70 (8.99)	− 3.913 (48)	0.000***
Verbal aggression	14.74 (2.73)	14.83 (3.74)	− 0.093 (48)	0.926
Anger	14.37 (3.96)	19.09 (5.82)	− 3.393 (48)	0.001**
Hostility	16.44 (4.90)	18.83 (5.52)	− 1.617 (48)	0.113
Total score	63.33 (12.24)	78.43 (21.55)	− 3.103 (48)	0.003**
Reactive–proactive aggression questionnaire (RPQ)				
Reactive aggression	6.56 (2.81)	13.85 (4.44)	− 7.224 (52)	0.000***
Proactive aggression	1.59 (2.83)	9.67 (5.19)	− 7.097 (52)	0.000***
Total score	8.15 (4.93)	23.52 (9.15)	− 7.682 (52)	0.000***
Psychopathy checklist-revised (PCL-R)				
Factor 1		6.64 (4.13)		
Factor 2		8.33 (3.85)		
Facet 1		2.63 (2.20)		
Facet 2		3.93 (2.37)		
Facet 3		3.78 (2.12)		
Facet 4		4.71 (2.90)		
Total score		16.15 (7.52)		
Brain volume estimates (mm^3^)				
TIV	1643.15 (136.64)	1603.48 (112.16)	1.166 (52)	0.249
GMV	743.19 (73.57)	710.85 (52.94)	1.854 (52)	0.069
WMV	565.11 (64.30)	556.85 (56.56)	0.501 (52)	0.618
CSF	334.81 (37.27)	334.74 (40.85)	0.007 (52)	0.994

Sample characteristics reporting mean value (*M*), standard deviation (SD), degrees of freedom (df) and *p* value (*p*) of the group comparisons (student’s *t* tests) between offenders (OF) and controls (HC)

*SUD* substance use disorder according to DSM-IV, *AQ* Buss & Perry Aggression questionnaire, *RPQ* reactive–proactive aggression questionnaire, *PCL-R* psychopathy checklist-revised, *TIV* total intracranial volume, *GMV* gray matter volume, *WMV* white matter volume, *CSF* cerebrospinal fluid; significance coefficient: *p < 0.05; **p < 0.01; ***p < 0.001

#### Correlation of trait aggression and psychopathy in offenders

As displayed in Table 2, correlational analyses between aggression and psychopathy sub-scales yielded throughout positive associations. The strongest effects (*p* < 0.001) were observed by correlating antisocial behavior (PCL-R facet 4) with physical (AQ) and reactive aggression (RPQ).

**Table 2:** Correlation of trait aggression and psychopathy in offenders

	Psychopathy checklist-revised (PCL-R)						
	Total	Factor 1	Factor 2	Facet 1	Facet 2	Facet 3	Facet 4
Aggression questionnaire (AQ)							
Total	0.509*	0.439*	0.508*	0.313	0.532*	0.165	0.628**
Physical	0.574**	0.503*	0.564**	0.346	0.616**	0.214	0.679***
Verbal	0.388	0.374	0.409	0.180	0.530*	0.114	0.554*
Anger	0.472*	0.478*	0.424	0.406	0.458*	0.126	0.534*
Hostility	0.304	0.139	0.360	0.111	0.222	0.094	0.421
Reactive–proactive aggression questionnaire (RPQ)							
Total	0.355	0.387	0.341	0.246	0.417*	− 0.122	0.597**
Reactive	0.340	0.326	0.351	0.254	0.314	− 0.168	0.660***
Proactive	0.336	0.406*	0.301	0.217	0.468*	− 0.071	0.490*

Bivariate correlation of trait aggression and psychopathy in offenders (significance coefficient: *p < 0.05; **p < 0.01; ***p < 0.001)

### MRI data results

#### Correlation of trait aggression and psychopathy with GMV in offenders

Whole brain multiple regression analyses in the offender group (*p* = 0.001, cluster forming threshold at *p*_(uncorrected)_ < 0.05) were performed with each sum- and sub-scale of the psychopathy and aggression tests separately (model 1) and revealed consistent negative correlations of both constructs with GMV (Table 3). Results of analyses including all sub-scales into statistical design (model 2) can be found in the supplement material.

**Table 3:** Negative correlations of gray matter volume (GMV) with aggression and psychopathy in offenders

Clusters (*k*)	Side	Anatomical region	*T*	Peak voxel			Effect sizes
				*X*	*Y*	*Z*	*R*^2^*/f*^2^
Negative correlation of GMV × psychopathy (PCL-R total score) in offenders							
275	R	Superior frontal gyrus	4.86	20	51	12	0.570/1.326
			4.66	23	53	2	
Negative correlation of GMV × impulsive–antisocial behavior (PCL-R factor 2) in offenders							
553	R	Superior frontal gyrus	5.82	21	51	11	0.677/2.097
			5.07	23	56	3	
		Middle frontal gyrus	5.01	27	57	5	
		Superior orbital gyrus	3.88	18	60	− 5	
			3.69	29	66	− 3	
371	R	Hippocampus	5.83	30	− 6	− 23	0.548/1.212
310	R	Inferior parietal lobule	5.08	51	− 53	45	0.527/1.114
Negative correlation of GMV × antisocial behavior (PCL-R facet 4) in offenders							
1702	R	Middle temporal gyrus	5.70	62	− 35	2	0.572/1.335
			5.44	60	− 41	2	
			4.51	54	− 30	− 9	
			3.81	51	− 23	− 15	
			3.70	63	− 17	− 17	
			3.68	69	− 17	− 20	
		Superior temporal gyrus	4.74	51	− 23	− 3	
			4.47	44	− 29	− 8	
701	L	Inferior parietal lobule	5.49	− 56	− 48	36	0.623/1.651
569	R	Superior frontal gyrus	7.19	23	56	3	0.697/2.301
			5.34	29	56	12	
		Superior frontal gyrus orbital	3.88	29	66	− 3	
			3.83	30	65	− 2	
Negative correlation of GMV x reactive aggression (RPQ) in offenders							
502	R	Middle temporal gyrus	4.38	53	− 27	− 11	0.408/0.690
		Superior temporal gyrus	3.72	45	− 18	− 8	

Significant results from voxel-by-voxel whole brain multiple regression analyses of GMV with sub-scale values of PCL-R and RPQ in criminal offenders at *p *= 0.001 significance level with a cluster-defining threshold at cluster-level *p*_(uncorrected)_< 0.05. No other sub-scales of the aggression and psychopathy measures correlated significantly with GMV in offenders. Cluster sizes in voxels (*k*), *T* values (*T*) and MNI coordinates (*x y z*) of cluster peaks are presented. Regions were defined by the WFU pickatlas for SPM (Maldjian et al. 2003). For each cluster effect sizes (*R*^2^ and Cohen’s *f*^2^) were estimated (Selya et al. 2012)

In summary, global psychopathy (PCL-R sum score) was negatively linked with GMV in prefrontal regions and this result was mainly determined by the negative correlation of the second factor of the PCL-R (impulsive–antisocial behavior) and more specifically with the fourth facet (antisocial behavior) of the scale. As depicted in detail in Table 3, antisocial behavior correlated negatively with GMV in right middle and superior temporal gyrus, right superior frontal and left inferior parietal regions.

**Fig. 1:**
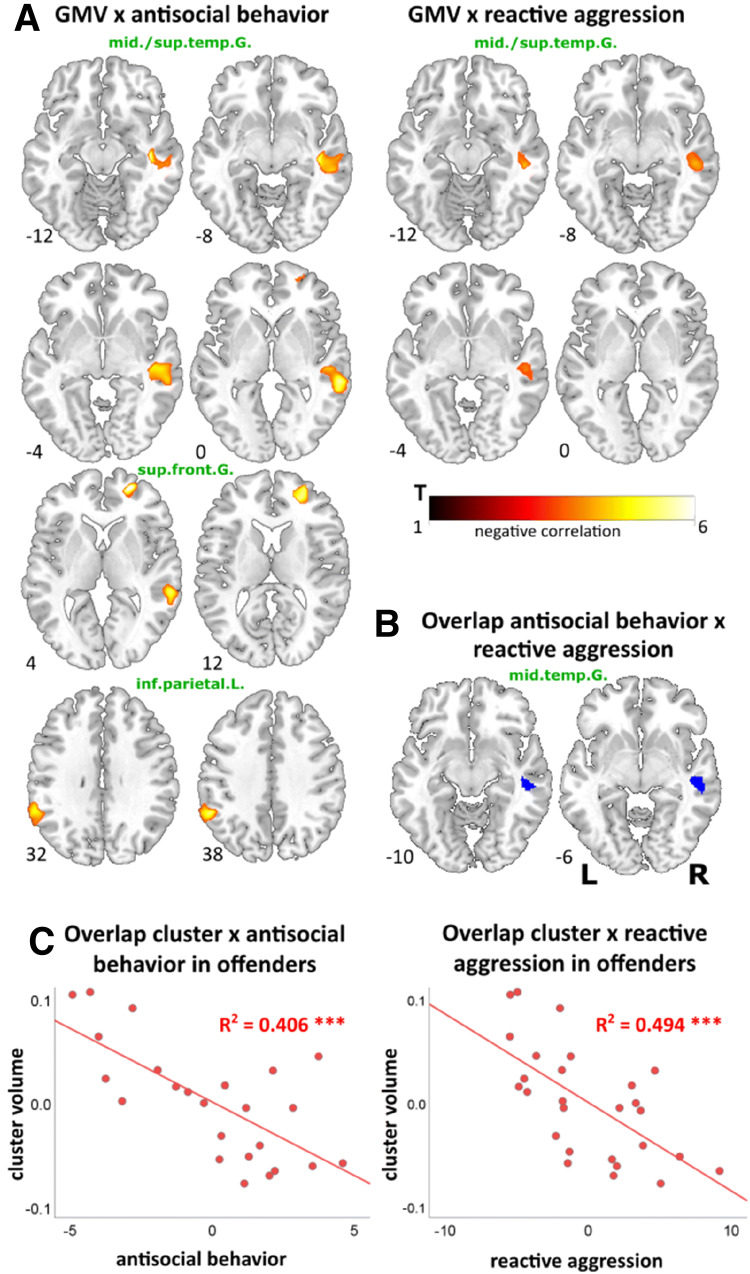
Negative correlations of gray matter volume (GMV) with aggression and psychopathy in offenders. **a** Negative correlations resulting from whole brain multiple regression analyses at *p* = 0.001 level with a cluster-defining threshold of *p*_(uncorrected)_ < 0.05 of GMV with antisocial behavior (PCL-R) reactive aggression (RPQ) in offenders, implementing total intracranial volume (TIV) and age as covariates. **b** Illustration of cluster emerging from the overlap of both results (RPQ reactive aggression × GMV and PCL-R antisocial behavior (facet 4) × GMV) comprising the right middle temporal gyrus. **c** Partial correlations of offenders individual GMV within the overlap cluster and PCL-R facet 4 (antisocial behavior) and RPQ reactive aggression scores revealed negative association of overlap cluster values with reactive aggression and antisocial behavior in offenders (controlled for TIV and age), ***p < 0.001

Only one sub-scale of the trait aggression scales correlated significantly with the offenders GMV. RPQ reactive aggression was negatively linked with GMV in the right middle and superior temporal gyrus (Table 3, Fig. 1a). No other sub-scales of the AQ, RPQ and PCL-R correlated significantly with the GMV of offenders.

#### Overlap of brain structure correlates of reactive aggression and antisocial behavior

One region in the right temporal cortex emerged in two in separate analyses. To identify the overlap, the results from the regression analyses of RPQ reactive aggression × GMV and PCL-R antisocial behavior (facet 4) × GMV were transformed into one unified MNI space and the MANGO function “create logical overlays” (Research Imaging Institue, UTHSCSA, 2018) was used. One cluster (952 mm^3^) in the right middle temporal gyrus (center of gravity *x y z*: 50 − 28 − 3) was found to be negatively correlated with both measures (Fig. 1b).

### Group comparison of gray matter volume (GMV)

No significant group difference in a voxel-by-voxel whole brain group comparison using TIV, IQ and age as control variables could be found, but a strong trend could be determined in the total GMV (*t*(52) = 1.854; *p* = 0.069, Table 1).

## Discussion

The aim of this study was to test the relationship between brain morphology and distinct aspects of psychopathic and aggressive behavior. In a cohort of 27 male criminal offenders less gray matter was found in the superior frontal gyrus with increasing psychopathy. This result was exclusively borne by the antisocial behavior facet of psychopathy, after no other sub-scales of the PCL-R could be significantly linked to brain structure specifics. Moreover, antisocial behavior of the offenders was associated with volume reductions in right superior frontal gyrus, right middle and superior temporal regions, and in left inferior parietal lobe. Particularly to highlight are the strongly pronounced effects in the right temporal lobe, which also became evident by a correlational analysis of offenders’ brain structure with reactive aggression. Also, no differences in gray matter volume between offenders and controls could be found.

The first main finding of this study was the inverse correlation between global psychopathy and gray matter volume. The higher the PCL-R sum scores of the offenders were, the less GMV was found in the superior parts of the prefrontal cortex. This result is in line with existing literature, linking global psychopathy with particular prefrontal gray matter volume reductions (see Pujol et al. 2018 for review), albeit positive correlations between PCL-R sum scores and prefrontal volumes were also reported (Korponay et al. 2017; Lam et al. 2017).

In a second step, and after correlating each of the PCL-R sub-scale scores with GMV, it became evident that the result of the correlation of global psychopathy with GMV was driven by one single sub-scale of the PCL-R. With increasing PCL-R facet 4 scores, i.e. severity of antisocial behavior, greater volume reductions were found in the right superior frontal gyrus, the right middle and superior temporal regions and the left inferior parietal lobe. The few studies that addressed comparable analyses, reported positive correlations of PCL-R facet 4 with basal ganglia structures volumes such as the caudate (Schiffer et al. 2011) or the lenticular nucleus (Glenn et al. 2010), whereas amygdala volume could be negatively linked with antisocial behavior. Mixed results were reported in prefrontal areas (Cope et al. 2014). For the implementation of these results into the existing body of research, it is necessary to consider that most of the mentioned studies performed regions of interest analyses and that only Cope et al. (2014) reported a regression analysis of the PCL-R facet scores with the whole brain gray matter volume.

The interpretation of volumetric results must be incorporated under the premise that morphological alterations are not equivalent to the functionality of the particular brain regions. Nevertheless, we will attempt to integrate our morphological results into broader neural network theories of human behavior and further try to bridge gray matter atrophies in cognitive control networks with antisocial behavior.

The finding that with increasing antisocial behavior tissue volumes are reduced in regions belonging to a fronto-temporo-parietal network, suggests a link between cognitive control and antisocial behavior. The functionality of this network is associated with high cognitive functioning, intelligence and mental flexibility (Marek and Dosenbach 2018; Petersen and Posner 2012) and disturbances have been reported in patients with impulsive–compulsive behavior (Tessitore et al. 2017) and antisocial personality disorder or psychopathy (Blair 2013, 2016; Raine 2018).

Above all, a prerequisite for all social behavior is a person’s ability to make assumptions about internal and mental states of another person, to predict others feelings, intentions, ideas and opinions. These social skills are summarized by the concept of “theory of mind” (ToM) and are often impaired in psychopathic or aggressive populations (Raine 2018; Stietz et al. 2019; Wai and Tiliopoulos 2012). The two dimensions of this construct, cognitive ToM (i.e. understanding others beliefs, intentions and motivations) and affective ToM (i.e. understanding others feelings, empathy) interestingly correlate differently with distinct features of psychopathy and aggression. On the one hand, emotionally detached psychopaths (PCL-R factor 1) showed less empathy (affective ToM), while no deficits in tasks assessing cognitive ToM were observed (Blair et al. 1996; Decety et al. 2013; Wai and Tiliopoulos 2012; Winter et al. 2017), low levels of cognitive ToM were linked with high reactive aggression (Renouf et al. 2010; Stellwagen and Kerig 2018), high affective ToM levels could be associated with proactive aggression in children (Renouf et al. 2010).

Coming back to our results, we found a negative link between antisocial behavior with tissue volume in regions overlapping with the cognitive ToM network, such as dorsomedial parts of the prefrontal cortex (i.e. the superior frontal gyrus), the tempo-parietal junction (i.e. inferior parietal regions) as well as in the middle temporal gyrus. Regions involved in the affective ToM network, that have been reported to be active during the contagion of positive emotions and empathy, such as the supramarginal gyrus, dorsolateral prefrontal and medial orbito-frontal cortex, anterior insula, ventral striatum and anterior medial cingulate cortex did not correlate significantly with any of the aggression or psychopathy measures in our study (Kanske et al. 2015; Stietz et al. 2019).

The finding, that regions of the fronto-temporo-parietal network, exhibit lower GMV the more antisocial behavior the participants were, underlines the assumption that different aspects of psychopathy and aggression are related to the two ToM dimensions. Hence we state that brain regions crucially involved in especially the inability to cognitively understand another persons’ intentions and behaviors are reduced in antisocial offenders, while brain regions involved in affective perspective taking were not linked with a psychopathy or aggression in our cohort.

Apart from theory of mind, the fronto-temporo-parietal network is further involved in information processing, and in particular in attention (Vossel et al. 2014). Neural networks attributed to higher attentional processes were allocated in primarily lateral and dorsomedial prefrontal, temporal and parietal regions. A well-established theory of social interaction deficits postulates that psychopaths have difficulties to continuously update incoming situational information when once being engaged into goal-oriented behavior (Blair 2013). This “response set modulation hypothesis” suggests that psychopaths often involuntarily neglect especially affective information (“bottle neck theory”), particularly if they are not relevant for the targeted outcome of the situation. These disruptions in selective attention processes might originate in corrupted top-down inhibitory quality of cortical regions on limbic neural activity. Interestingly we find GMV reductions in those cortical regions relevant for selective attention with increasing antisocial behavior. Hence we conclude, that problems in attention setting, shifting and updating might be linked with the antisocial behavior facet of the PCL-R.

Lastly, and with regard to our findings we want to highlight the role of the right temporal lobe and discuss its function in terms of aggressive behavior from a clinical point of view. The relationship between temporal gray matter reduction and especially impulsive aggressive behavior has previously been supported by clinical studies. In neurological or neurodegenerative disorders, such as epilepsy and dementia, aggressive behavior frequently occurs in the course of the disease. Phenomenologically, some of these patients were reported to display aggressive behavior, anger outbursts or the inability to hold their temper (see Levenson et al. 2014; Müller-Spahn 2003 for review). Interestingly, diseases accompanied by aggressive symptoms involve neurodegenerations or dysfunctions of particularly temporal regions. For example, patients suffering from Alzheimer’s disease, temporal lobe epilepsy or temporal brain lesions are often reported to exhibit inappropriate or aggressive behavior, which is often correlated with abnormal functioning of, or tissue loss in the right temporal regions (Adolphs et al. 2000; Chan et al. 2009; Haller and Kruk 2006).

### Group comparison

Despite both sample groups differed in education, substance use, trait aggressiveness and criminal history, strong trends but no significant group differences in GMV were found in a whole brain analysis after controlling for TIV, age and IQ. This result contradicts our hypothesis and findings of previous studies that reported reduced regional GMV in offenders compared to controls. Of the 35 studies included in a recent review (Lamsma et al. 2017), only four papers reported comparable designs to our study (i.e. VBM in group comparisons between forensic and healthy sample without comorbidities other than SUD). The majority of these studies revealed GMV reductions in offenders compared to controls in prefrontal areas, including superior, medial, lateral and inferior parts of the frontal lobe (Bertsch et al. 2013; Contreras-Rodríguez et al. 2015; Gregory et al. 2012), temporal regions (Contreras-Rodríguez et al. 2015; Gregory et al. 2012), precuneus (Bertsch et al. 2013; Contreras-Rodríguez et al. 2015), pre- and postcentral gyri (Bertsch et al. 2013; Gregory et al. 2012) and anterior and medial parts of the cingulate cortex (Bertsch et al. 2013). Subcortical atrophies in the amygdala-hippocampal region (Bertsch et al. 2013; Contreras-Rodríguez et al. 2015; Schiffer et al. 2011) and the basal ganglia (Schiffer et al. 2011) were also described.

Comparisons with other studies are impeded by two major aspects. First, it is crucial to state that most of the reported studies analyzed regional gray matter volumes (ROI) rather than applied a whole brain approach which might results in diminished comparability of the results. Secondly, our null-results might be related to the characteristics of our cohorts. Although all offenders were convicted for at least one violent crime and displayed significantly higher levels of trait aggression compared to the controls, test scores of the PCL-R, AQ and RPQ did not reach the top percentile of the scales normatives.

## Limitation

In terms of limitation of our study, we would like to point out that differences between the two sample groups could not be prevented and should be taken into account when interpreting the results of the group comparison. Offenders were characterized by significant lower verbal IQ, less educational years and higher prevalence of substance use disorders compared to controls. Despite efforts to match the control group for IQ, education and substance use disorders, we were not able to recruit a non-criminal control group fulfilling these criteria. Therefore, possible influences of intelligence and substance use on brain morphology cannot be fully ruled out, since both could be associated with GM reductions in widespread cortical regions (Goriounova and Mansvelder 2019; Haier et al. 2004; Stoychev 2019). However, since the main results of the study were based on correlational analysis within the group of offenders and since this group was fairly homogeneously constituted with regard to IQ and SUD, the group differences do not interfere with the main results.

The assessment of psychopathy using the PCL-R was carried out only in the offenders group, which does not allow a direct comparison of the findings of the correlational analyses of this trait with GMV of both sample groups. However, the rational to only perform the PCL-R in offenders can be found in the nature of the scale itself. Due to the fact that the PCL-R is an elaborated clinical interview which was developed primarily for the diagnosis of psychopathy and, therefore, targeting a forensic group, there was no reason to apply this instrument in the control group. Previous validation studies described very low levels of psychopathy in community samples, which could result in insufficient variance necessary for correlational analysis.

Another shortcoming of this study could be seen the small number of cases. With 27 participants in each group, the sample appears to be quite small in the field of MRI research. However, it should be noted that in the context of a German forensic sample, the offender cohort was large. Also, effect sizes of significant correlations revealed affirmed statistical power. MRI research in criminal offenders is particularly effortful and as described in the methods section, only 40% of the offenders explicitly recruited to meet the study criteria did actually fulfill them.

## Conclusions

With regard to our findings, we propose that psychopathy and more specifically, its antisocial aspects are associated with GMV reductions in areas involved in social information processing and cognitive functioning, including prefrontal, temporal and parietal regions. Here the results in the temporal lobe are of particular importance, since they could be replicated by the correlation of GMV with reactive aggression and could often be linked with aggressive behavior in clinical cohorts. Another important aspect of our study, which has received scarce attention in previous neuroimaging research, is the fact that different aspects of psychopathic or aggressive behavior, measured with respective sub-scales, correlate differently with whole brain gray matter volume. As mentioned above, we provided evidence that the overall result of the correlation of PCL-R sum scores with GMV was mainly driven by one single facet of the scale, i.e. antisocial behavior. These results indicate that some specific sub-factors of psychopathic and aggressive behavior bare a much greater influence than others and hence determine the overall results. In our case trait components of psychopathy and aggression, i.e. emotional detachment or proactive aggression are not associated with brain morphology, whereas the behavioral aspects, such as antisocial behavior and reactive aggression are significantly linked to gray matter reductions. Only a few studies have taken this approach of analyzing sub-facet and their contribution to the overall result separately and hence comparable studies are scarce which impedes the integration of our results into existing research and emphasizes the need for future research to improve the coherence of existing results. To expand the understanding of the relationship between brain structure and specific components of aggressive and psychopathic behavior, future studies should be conducted with a similar approach as the experiment presented here.

## Electronic supplementary material

Below is the link to the electronic supplementary material.

Supplementary material 1 (DOCX 33 kb)
