# Supplementary material for: Morphology of the criminal brain: gray matter reductions are linked to antisocial behavior in offenders
Source: Brain Struct Funct. 2020 Jun 26;225(7):2017–28. doi: 10.1007/s00429-020-02106-6 (PMC7473962; doi:10.1007/s00429-020-02106-6)
Supplement: Supplementary file 1 — Supplementary material 1 (DOCX 33 kb) [file 429_2020_2106_MOESM1_ESM.docx]

Supplemental material

**Morphology of the Criminal Brain: Gray Matter Reductions are linked to Antisocial Behavior in Offenders**

Lena Hofhansel^1,2^, Carmen Weidler^1^, Mikhail Votinov^1,2^, Benjamin Clemens^1^, Adrian Raine^3^, Ute Habel^1,2^

1. Department of Psychiatry, Psychotherapy and Psychosomatics, Medical Faculty, RWTH Aachen University, Aachen, Germany; 2. Institute of Neuroscience and Medicine (INM-10), Research Center Jülich, Jülich, Germany, 3. Departments of Criminology, Psychiatry, and Psychology, University of Pennsylvania, Philadelphia, PA, USA

**S 1: Correlation of Trait Aggression and Psychopathy with GMV in Offenders (model 2)**

For the calculation of model 2, whole brain multiple regression analyses in the offender group (*p* = .001, cluster forming threshold at *p_(uncorrected)_* < .05) were performed including all sub-scales of respective measure (e.g. all four sub-scales of the AQ into one design) as well as TIV and age as variables of no interest. Results of the AQ (S1.1.), RPQ (S1.2.) and PCL-R (S1.3.) are reported below. Results of model 1 (analyses of respective sub-scales in separate designs) can be found in the main manuscript.

S1.1.: Model 2 Aggression Questionnaire (AQ) in offenders

| **Clusters (*k*)** | **Side** | **Anatomical region** |  | **Peak voxel** | | |
| --- | --- | --- | --- | --- | --- | --- |
|  |  |  | ***T*** | **X** | **Y** | **Z** |
| ***Positive correlation of GMV x physical aggression (AQ) in offenders***  n.s*.* | | | | | | |
| ***Negative correlation of GMV x physical aggression (AQ) in offenders*** | | | | | | |
| 1641 | L | Putamen | 6.69 | -27 | -8 | -6 |
|  |  |  | 6.26 | -30 | -15 | -3 |
|  |  | Parahippocampal gyrus | 5.12 | -12 | -14 | -26 |
|  |  | Pallidum | 4.64 | -20 | -6 | 5 |
| 1323 | R | Pallidum | 5.34 | 21 | -8 | 5 |
|  |  |  | 5.26 | 27 | -12 | -3 |
|  |  | Putamen | 5.20 | 26 | 0 | 0 |
|  |  |  | 4.73 | 27 | -9 | 14 |
|  |  | Hippocampus | 4.40 | 32 | -9 | -14 |
| ***Positive correlation of GMV x verbal aggression (AQ) in offenders*** | | | | | | |
| 348 | L | Parahippocampal gyrus | 6.41 | -12 | -14 | -27 |
|  |  |  | 5.68 | -15 | -12 | -33 |
| ***Negative correlation of GMV x verbal aggression (AQ) in offenders*** | | | | | | |
| n.s. |  |  |  |  |  |  |
| ***Positive correlation of GMV anger (AQ) in offenders*** | | | | | | |
| 243 | L | Cerebellum Crus 1 | 5.00 | -50 | -45 | -30 |
|  |  | Inferior temporal gyrus | 4.73 | -48 | -45 | -14 |
|  |  |  | 4.45 | -51 | -54 | -15 |
| ***Negative correlation of GMV x anger (AQ) in offenders*** | | | | | | |
| n.s. |  |  |  |  |  |  |
| ***Positive correlation of GMV x hostility (AQ) in offenders*** | | | | | | |
| 1172 | R | Putamen | 4.51 | 26 | 2 | 0 |
|  |  | Caudate | 4.34 | 15 | -2 | 24 |
|  |  | Pallidum | 4.18 | 23 | -8 | -3 |
| 942 | L | Pallidum | 5.14 | -21 | -3 | 2 |
|  |  | Putamen | 4.97 | -29 | -3 | 5 |
|  |  |  | 4.45 | -26 | -17 | 8 |
|  |  | Caudate | 4.46 | -11 | 18 | 12 |
|  |  |  | 3.90 | -14 | 12 | 18 |
|  |  |  | 3.84 | -17 | 9 | 21 |
|  |  |  | 3.82 | -18 | 11 | 17 |
| ***Negative correlation of GMV x hostility (AQ) in offenders*** | | | | | | |
| 1054 | L | Middle temporal pole | 4.46 | -48 | 17 | -36 |
| 363 | R | Cerebellum Crus 1 | 6.30 | 44 | -68 | -24 |
| 232 | L | Inferior temporal gyrus | 6.69 | -59 | -3 | -39 |
|  |  |  | 4.18 | -51 | -11 | -26 |

S1.2.: Model 2 Reactive-Proactive Aggression Questionnaire (AQ) in offenders

| **Clusters (*k*)** | **Side** | **Anatomical region** |  | **Peak voxel** | | |
| --- | --- | --- | --- | --- | --- | --- |
|  |  |  | ***T*** | **X** | **Y** | **Z** |

| ***Positive correlation of GMV x reactive aggression (RPQ) in offenders*** | | | | | | |
| --- | --- | --- | --- | --- | --- | --- |
| n.s. |  |  |  |  |  |  |
| ***Negative correlation of GMV x reactive aggression (RPQ) in offenders*** | | | | | | |
| n.s. |  |  |  |  |  |  |
| ***Positive correlation of GMV x proactive aggression (RPQ) in offenders*** | | | | | | |
| n.s. |  |  |  |  |  |  |
| ***Negative correlation of GMV x proactive aggression (RPQ) in offenders*** | | | | | | |
| 347 | L | Inferior temporal gyrus | 6.04 | -50 | -51 | -14 |

S1.3.: Model 2 Psychopathy Checklist – Revised (PCL-R) in offenders (four facets)

| **Clusters (*k*)** | **Side** | **Anatomical region** |  | **Peak voxel** | | |
| --- | --- | --- | --- | --- | --- | --- |
|  |  |  | ***T*** | **X** | **Y** | **Z** |

| ***Positive correlation of GMV x facet 1 (PCL-R) in offenders*** | | | | | | |
| --- | --- | --- | --- | --- | --- | --- |
| n.s. |  |  |  |  |  |  |
| ***Negative correlation of GMV x facet 1 (PCL-R) in offenders*** | | | | | | |
| n.s. |  |  |  |  |  |  |
| ***Positive correlation of GMV x facet 2 (PCL-R) in offenders*** | | | | | | |
| 231 | L | Superior parietal gyrus | 6.94 | -23 | -69 | 60 |
| ***Negative correlation of GMV x facet 2 (PCL-R) in offenders*** | | | | | | |
| n.s. |  |  |  |  |  |  |
| ***Positive correlation of GMV x facet 3 (PCL-R) in offenders*** | | | | | | |
| n.s. |  |  |  |  |  |  |
| ***Negative correlation of GMV x facet 3 (PCL-R) in offenders*** | | | | | | |
| n.s. |  |  |  |  |  |  |
| ***Positive correlation of GMV x facet 4 (PCL-R) in offenders*** | | | | | | |
| n.s. |  |  |  |  |  |  |
| ***Negative correlation of GMV x facet 4 (PCL-R) in offenders*** | | | | | | |
| 1708 | R | Middle temporal pole | 5.88 | 53 | -30 | -8 |
|  |  |  | 5.20 | 60 | -42 | 2 |
|  |  |  | 4.96 | 60 | -33 | 0 |
|  |  | Superior temporal gyrus | 5.72 | 56 | -21 | -2 |
|  |  |  | 5.54 | 45 | -9 | -12 |
|  |  |  | 4.82 | 44 | -23 | -2 |
| 413 | L | Supramarginal gyrus | 5.47 | -62 | -47 | 35 |
|  |  | Inferior parietal gyrus | 5.16 | -56 | -48 | 36 |
| 298 | R | Superior frontal gyrus | 5.79 | 29 | 51 | 12 |
|  |  |  | 5.64 | 23 | 56 | 3 |
| 234 | L | Rectal gyrus | 4.70 | -2 | 24 | -17 |
|  |  | Anterior cingulate cortex | 4.43 | -8 | 33 | -9 |

**S 2: Correlation of Trait Aggression with GMV in Controls (model 1&2)**

For model 1 whole brain multiple regression analyses in the control group (*p* = .001, cluster forming threshold at *p_(uncorrected)_* < .05) were performed for all sub-scales of respective measure separately (see S2.1 for AQ scores and S2.2 for RPQ scores). In model 2, all sub-scales of a measure were included into one single statistical design (S2.3. for AQ scores, S2.4. for RPQ scores). In both approaches TIV and age were used as variables of no interest. Results for AQ and RPQ are reported below.

S 2.1.: Model 1 Aggression Questionnaire (AQ) in controls

| **Clusters (*k*)** | **Side** | **Anatomical region** |  | **Peak voxel** | | |
| --- | --- | --- | --- | --- | --- | --- |
|  |  |  | ***T*** | **X** | **Y** | **Z** |

| ***Positive correlation of GMV x AQ total score in controls*** | | | | | | |
| --- | --- | --- | --- | --- | --- | --- |
| n.s. |  |  |  |  |  |  |
| ***Negative correlation of GMV x AQ total score in controls*** | | | | | | |
| 273 | R | Postcentral gyrus | 4.85 | 30 | -39 | 57 |
| ***Positive correlation of GMV x physical aggression (AQ) in controls*** | | | | | | |
| n.s. |  |  |  |  |  |  |
| ***Negative correlation of GMV x physical aggression (AQ) in controls*** | | | | | | |
| n.s. |  |  |  |  |  |  |
| ***Positive correlation of GMV x verbal aggression (AQ) in controls*** | | | | | | |
| n.s. |  |  |  |  |  |  |
| ***Negative correlation of GMV x verbal aggression (AQ) in controls*** | | | | | | |
| n.s. |  |  |  |  |  |  |
| ***Positive correlation of GMV x anger (AQ) in controls*** | | | | | | |
| n.s. |  |  |  |  |  |  |
| ***Negative correlation of GMV x anger (AQ) in controls*** | | | | | | |
| n.s. |  |  |  |  |  |  |
| ***Positive correlation of GMV x hostility (AQ) in controls*** | | | | | | |
| 362 | L | Middle temporal gyrus | 4.89 | -63 | -36 | -3 |
|  |  |  | 4.36 | -71 | -38 | -9 |
|  |  |  | 4.32 | -71 | -35 | -6 |
| ***Negative correlation of GMV x hostility (AQ) in controls*** | | | | | | |
| n.s. |  |  |  |  |  |  |

S 2.2.: Model 1 Reactive-Proactive Aggression Questionnaire in controls

| **Clusters (*k*)** | **Side** | **Anatomical region** |  | **Peak voxel** | | |
| --- | --- | --- | --- | --- | --- | --- |
|  |  |  | ***T*** | **X** | **Y** | **Z** |

| ***Positive correlation of GMV x RPQ total score in controls*** | | | | | | | | | | | | |
| --- | --- | --- | --- | --- | --- | --- | --- | --- | --- | --- | --- | --- |
| 422 | | R | Middle occipital gyrus | | 5.54 | | | 33 | | -92 | | 0 |
|  |  |  |  |  | 3.83 | | | 41 | | -84 | | 3 |
| 405 | | L | Middle temporal pole | | 4.67 | | | -33 | | 18 | | -35 |
|  |  |  |  |  | 4.53 | | | -36 | | 26 | | -36 |
|  |  |  |  |  | 4.28 | | | -41 | | 24 | | -38 |
|  |  |  |  |  | 3.67 | | | -23 | | 18 | | -39 |
| ***Negative correlation of GMV x RPQ total score in controls*** | | | | | | | | | | | | |
| n.s. | |  |  | |  | | |  | |  | |  |
| ***Positive correlation of GMV x reactive aggression (RPQ) in controls*** | | | | | | | | | | | | |
| n.s. |  | | |  | |  |  | |  | |  |  |
| ***Negative correlation of GMV x reactive aggression (RPQ) in controls*** | | | | | | | | | | | | |
| n.s. |  |  |  |  |  |  |  |  |  |  |  |  |
| ***Positive correlation of GMV x proactive aggression (RPQ) in controls*** | | | | | | | | | | | | |
| 2841 | | L | Middle temporal pole | | 7.89 | | | -33 | | 18 | | -35 |
|  |  |  |  |  | 7.55 | | | -36 | | 26 | | -36 |
|  |  |  |  |  | 7.40 | | | -41 | | 24 | | -38 |
|  |  |  | Superior temporal pole | | 5.60 | | | -48 | | 11 | | -21 |
|  |  |  |  |  | 4.01 | | | -47 | | 21 | | -21 |
|  |  |  |  |  | 3.79 | | | -54 | | 15 | | -8 |
|  |  |  | Superior temporal gyrus | | 3.26 | | | -48 | | 5 | | -5 |
| 488 | | L | Superior orbital frontal gyrus | | 6.31 | | | -8 | | 36 | | -30 |
|  |  |  |  |  | 6.29 | | | -11 | | 27 | | -29 |
| 381 | | L | Superior frontal gyrus | | 5.25 | | | -26 | | 48 | | 20 |
| ***Negative correlation of GMV x proactive aggression (RPQ) in controls*** | | | | | | | | | | | | |
| n.s. |  |  |  |  |  |  |  |  |  |  |  |  |

S 2.3.: Model 2 Aggression Questionnaire (AQ) in controls

| **Clusters (*k*)** | **Side** | **Anatomical region** |  | **Peak voxel** | | |
| --- | --- | --- | --- | --- | --- | --- |
|  |  |  | ***T*** | **X** | **Y** | **Z** |

| ***Positive correlation of GMV x physical aggression (AQ) in controls*** | | | | | | |
| --- | --- | --- | --- | --- | --- | --- |
| n.s. |  |  |  |  |  |  |
| ***Negative correlation of GMV x physical aggression (AQ) in controls*** | | | | | | |
| n.s. |  |  |  |  |  |  |
| ***Positive correlation of GMV x verbal aggression (AQ) in controls*** | | | | | | |
| n.s. |  |  |  |  |  |  |
| ***Negative correlation of GMV x verbal aggression (AQ) in controls*** | | | | | | |
| n.s. |  |  |  |  |  |  |
| ***Positive correlation of GMV x anger (AQ) in controls*** | | | | | | |
| n.s. |  |  |  |  |  |  |
| ***Negative correlation of GMV x anger (AQ) in controls*** | | | | | | |
| n.s. |  |  |  |  |  |  |
| ***Positive correlation of GMV x hostility (AQ) in controls*** | | | | | | |
| n.s. |  |  |  |  |  |  |
| ***Negative correlation of GMV x hostility (AQ) in controls*** | | | | | | |
| n.s. |  |  |  |  |  |  |

S 2.4.: Model 2 Reactive-Proactive Aggression Questionnaire in controls

| **Clusters (*k*)** | **Side** | **Anatomical region** |  | **Peak voxel** | | |
| --- | --- | --- | --- | --- | --- | --- |
|  |  |  | ***T*** | **X** | **Y** | **Z** |

| ***Positive correlation of GMV x reactive aggression (RPQ) in controls*** | | | | | | |
| --- | --- | --- | --- | --- | --- | --- |
| 382 | R | Cuneus | 5.55 | 17 | -87 | 38 |
| ***Negative correlation of GMV x reactive aggression (RPQ) in controls*** | | | | | | |
| 267 | R | Superior medial frontal gyrus | 4.44 | 5 | 33 | 62 |
| ***Positive correlation of GMV x proactive aggression (RPQ) in controls*** | | | | | | |
| 2952 | L | Middle temporal pole | 7.17 | -35 | 20 | -35 |
|  |  | Superior temporal pole | 5.73 | -48 | 9 | -18 |
|  |  |  | 3.58 | -45 | 23 | -21 |
|  |  |  | 3.51 | -48 | 5 | -6 |
|  |  | Parahippocampal gyrus | 5.14 | -27 | -15 | -33 |
|  |  | Insula | 4.77 | -45 | 12 | -9 |
| 729 | L | Superior orbitofrontal gyrus | 5.70 | -11 | 26 | -29 |
|  |  |  | 5.60 | -8 | 38 | -30 |
|  |  |  | 4.40 | -15 | 51 | -24 |
| 446 | L | Middle frontal gyrus | 4.83 | -32 | 60 | 8 |
|  |  |  | 4.28 | -36 | 54 | 6 |
|  |  |  | 4.21 | -33 | 59 | 20 |
|  |  |  | 4.20 | -39 | 56 | 20 |
|  |  | Superior frontal gyrus | 4.26 | -26 | 50 | 20 |
| 319 | L | Inferior frontal gyrus, pars triangularis | 6.91 | -45 | 24 | 14 |
| ***Negative correlation of GMV x proactive aggression (RPQ) in controls*** | | | | | | |
| n.s. |  |  |  |  |  |  |

**S 3: Correlation of Trait Aggression with GMV in Offenders and Controls (model 1&2)**

For model 1 whole brain multiple regression analyses in the entire sample (*p* = .001, cluster forming threshold at *p_(uncorrected)_* < .05) were performed for all sub-scales of respective measure separately. In model 2, all sub-scales of a measure were included into one single statistical design. In both approaches TIV and age were used as variables of no interest. Results for AQ and RPQ are reported below.

S.3.1. Model 1 Aggression Questionnaire (AQ) in offenders and controls combined

| **Clusters (*k*)** | **Side** | **Anatomical region** |  | **Peak voxel** | | |
| --- | --- | --- | --- | --- | --- | --- |
|  |  |  | ***T*** | **X** | **Y** | **Z** |

| ***Positive correlation of GMV x AQ total score in entire sample*** | | | | | | |
| --- | --- | --- | --- | --- | --- | --- |
| n.s. |  |  |  |  |  |  |
| ***Negative correlation of GMV x AQ total score in entire sample*** | | | | | | |
| n.s. |  |  |  |  |  |  |
| ***Positive correlation of GMV x physical aggression (AQ) entire sample*** | | | | | | |
| n.s. |  |  |  |  |  |  |
| ***Negative correlation of GMV x physical aggression (AQ) entire sample*** | | | | | | |
| n.s. |  |  |  |  |  |  |
| ***Positive correlation of GMV x verbal aggression (AQ) entire sample*** | | | | | | |
| n.s. |  |  |  |  |  |  |
| ***Negative correlation of GMV x verbal aggression (AQ) entire sample*** | | | | | | |
| n.s. |  |  |  |  |  |  |
| ***Positive correlation of GMV x anger (AQ) in entire sample*** | | | | | | |
| n.s. |  |  |  |  |  |  |
| ***Negative correlation of GMV x anger (AQ) in entire sample*** | | | | | | |
| 771 | L | Middle orbitofrontal gyrus | 5.21 | -32 | 57 | -8 |
| ***Positive correlation of GMV x hostility (AQ) in entire sample*** | | | | | | |
| n.s. |  |  |  |  |  |  |
| ***Negative correlation of GMV x hostility (AQ) in entire sample*** | | | | | | |
| n.s. |  |  |  |  |  |  |

S 3.2.: Model 1 Reactive-Proactive Aggression Questionnaire in offenders and controls combined

| **Clusters (*k*)** | **Side** | **Anatomical region** |  | **Peak voxel** | | |
| --- | --- | --- | --- | --- | --- | --- |
|  |  |  | ***T*** | **X** | **Y** | **Z** |

| ***Positive correlation of GMV x RPQ total score in entire sample*** | | | | | | |
| --- | --- | --- | --- | --- | --- | --- |
| n.s. |  |  |  |  |  |  |
| ***Negative correlation of GMV x RPQ total score in entire sample*** | | | | | | |
| n.s. |  |  |  |  |  |  |
| ***Positive correlation of GMV x reactive aggression (RPQ) in entire sample*** | | | | | | |
| n.s. | | | | | | |
| ***Negative correlation of GMV x reactive aggression (RPQ) in entire sample*** | | | | | | |
| n.s. | | | | | | |
| ***Positive correlation of GMV x proactive aggression (RPQ) in entire sample*** | | | | | | |
| n.s. |  |  |  |  |  |  |
| ***Negative correlation of GMV x proactive aggression (RPQ) in entire sample*** | | | | | | |
| n.s. |  |  |  |  |  |  |

S 3.3.: Model 2 Aggression Questionnaire (AQ) in offenders and controls combined

| **Clusters (*k*)** | **Side** | **Anatomical region** |  | **Peak voxel** | | |
| --- | --- | --- | --- | --- | --- | --- |
|  |  |  | ***T*** | **X** | **Y** | **Z** |

| ***Positive correlation of GMV x physical aggression (AQ) in entire sample*** | | | | | | |
| --- | --- | --- | --- | --- | --- | --- |
| n.s. |  |  |  |  |  |  |
| ***Negative correlation of GMV x physical aggression (AQ) in entire sample*** | | | | | | |
| n.s. |  |  |  |  |  |  |
| ***Positive correlation of GMV x verbal aggression (AQ) in entire sample*** | | | | | | |
| n.s. |  |  |  |  |  |  |
| ***Negative correlation of GMV x verbal aggression (AQ) in entire sample*** | | | | | | |
| 398 | L | Parahippocampal gyrus | 4.07 | -23 | -6 | -29 |
|  |  |  | 4.04 | -15 | 5 | -26 |
| ***Positive correlation of GMV x anger (AQ) in entire sample*** | | | | | | |
| n.s. |  |  |  |  |  |  |
| ***Negative correlation of GMV x anger (AQ) in entire sample*** | | | | | | |
| n.s. |  |  |  |  |  |  |
| ***Positive correlation of GMV x hostility (AQ) in entire sample*** | | | | | | |
| 441 | R | Thalamus | 3.91 | 17 | -21 | -2 |
|  |  |  | 3.88 | 12 | -30 | -3 |
|  |  |  | 3.54 | 11 | -23 | -3 |
| 382 | L | Angular gyrus | 4.52 | -47 | -66 | 24 |
|  |  | Middle temporal gyrus | 3.85 | -51 | -69 | 12 |
| ***Negative correlation of GMV x hostility (AQ) in entire sample*** | | | | | | |
| n.s. |  |  |  |  |  |  |

S 3.4.: Model 2 Reactive-Proactive Aggression Questionnaire in offenders and controls combined

| **Clusters (*k*)** | **Side** | **Anatomical region** |  | **Peak voxel** | | |
| --- | --- | --- | --- | --- | --- | --- |
|  |  |  | ***T*** | **X** | **Y** | **Z** |

| ***Positive correlation of GMV x reactive aggression (RPQ) in entire sample*** | | | | | | |
| --- | --- | --- | --- | --- | --- | --- |
| 384 | L | Calcarine gyrus | 3.94 | -15 | -56 | 8 |
|  |  |  | 3.50 | -18 | -72 | 12 |
| ***Negative correlation of GMV x reactive aggression (RPQ) in entire sample*** | | | | | | |
| 527 | L | Parahippocampal gyrus | 3.86 | -29 | -3 | -33 |
| ***Positive correlation of GMV x proactive aggression (RPQ) in entire sample*** | | | | | | |
| n.s. |  |  |  |  |  |  |
| ***Negative correlation of GMV x proactive aggression (RPQ) in entire sample*** | | | | | | |
| 379 | R | Lingual gyrus | 4.47 | 20 | -89 | -2 |
|  |  | Calcarine gyrus | 4.37 | 14 | -93 | 5 |
|  |  | Inferior occipital gyrus | 3.73 | 32 | -86 | -8 |
